# Toward the Goal of Leaving No One Behind: Orthostatic Dysregulation

**DOI:** 10.31662/jmaj.2022-0225

**Published:** 2023-05-10

**Authors:** Kazuma Shinno, Shinichiro Nagamitsu

**Affiliations:** 1 Department of Pediatrics, National Hospital Organization Saitama Hospital, Saitama, Japan; 2 Department of Pediatrics, Fukuoka University, Fukuoka, Japan

**Keywords:** Orthostatic Dysregulation, COVID-19, Digital Therapeutics, Web Application, Psychosomatic Disease, School Refusal

## Abstract

Orthostatic dysregulation (OD), a common psychosomatic disorder in children, is caused by circulation disturbance resulting from autonomic imbalance. OD is a significant public health threat due to its association with school refusal and depression in children. During the COVID-19 pandemic, many children suffered from school closures, lack of exercise, smartphone addiction, and school refusal. The COVID-19 pandemic made it much more difficult to deliver existing approaches to patients and families with OD and increased the risk of prolonged and severe OD. In response, web-based digital health solutions are expected to support patients and families. Digital therapeutics for OD can not only deliver established treatments online, such as pharmacotherapy and behavioral therapy but also provide new interventions, such as regular mental health programs led by clinical psychologists. It is necessary to keep in mind that digital therapeutics are not intended to replace established treatments, but rather to supplement them and provide additional support. However, most research on OD has been conducted in Japan. Therefore, to provide unique findings from Japan, it is important to conduct further epidemiological research using large-scale databases in the real world and reveal the clinical characteristics and detailed epidemiology of OD, leading to the development of novel treatments.

## Orthostatic Dysregulation from a Public Health Perspective

Orthostatic dysregulation (OD) is a common psychosomatic disease in children characterized by circulation disturbance triggered by autonomic imbalance. Patients with OD may present various symptoms, including headache, nausea, loss of appetite, and dizziness. In Japan, OD is estimated to affect approximately 700,000 children: 5% of elementary school and 10% of junior and senior high school students. It is also a leading cause of school refusal, with 30%-40% of cases attributed to OD ^(1)^. Most children with OD have a good prognosis and recover from mild OD, but severe OD can lead to long-term truancy, sleep disorders, and psychiatric diseases. In addition, 10% of patients with severe OD develop depression, which can take several years to recover from and impact their schooling and employment. The COVID-19 pandemic has increased the risk of prolonged and severe OD, including school closures, deconditioning, smartphone addiction, and fear of infection ^(2)^. During the pandemic, the number of children who refused to go to school for a long term reached a record high of 245,000 per year ^(3)^. Overall, OD is a significant public health concern due to its impact on school attendance and mental health.

## Established Approaches

According to the guidelines of the Japanese Society of Psychosomatic Pediatrics, patient education and non-pharmacological treatment should be provided in all cases of OD ^(1)^. OD is classified into three levels of severity: mild, moderate, and severe. Depending on the severity, additional treatments may be recommended, including pharmacotherapy, psychotherapy, cooperation with school teachers, and environmental control around the patients, as well as support from friends and family ^(1)^. A lack of understanding about OD is a significant problem, as it can lead to patients being regarded as falsely ill or lazy. This can further exacerbate clinical symptoms and add psychological stress. During the COVID-19 pandemic, patients may have difficulty accessing established approaches due to their voluntary absence from school and reluctance to seek medical care in hospitals to avoid infection. According to the report by the Japan Pediatric Society, the total number of pediatric outpatients in hospitals across Japan reduced to 54.6% during the COVID-19 pandemic ^(4)^. On the other hand, 26% of all medical facilities reported an increase in the number of pediatric outpatients who presented with school refusal ^(4)^.

## Emerging Issues during the COVID-19 Pandemic

During the COVID-19 pandemic, several issues related to OD have emerged that can be discussed from three standpoints: the patients and their parents, the educators, and the health care providers.

First, patients and their parents suffering from OD may be less likely to use existing interventions, leading to a worsening of severity and longer-lasting symptoms. In addition, even if OD was triggered by the COVID-19 pandemic, they may not recognize it and be overlooked due to a lack of knowledge. The COVID-19 pandemic drastically changed the environment surrounding children, including school closures, limited social interactions, reduced physical activity, and increased screen time. These changes may contribute to physical deconditioning, leading to the development, severity, and prolongation of OD.

Second, for educators, school closures and increased voluntary absences have made face-to-face communication more difficult, potentially contributing to misunderstandings and stigma about patients. Educators have had to focus on preventing the spread of infection among their classmates and have been sensitive to the possibility of COVID-19. As a result, they were obliged to prohibit children with COVID-19-like symptoms from attending school since it was quite difficult to distinguish between OD and COVID-19 during the pandemic.

Third, medical professionals have had much more difficulty providing established treatments to patients with OD. In addition to patients avoiding seeking health care services, medical professionals may have been forced to postpone clinical interventions in hospitals if patients developed COVID-19-related symptoms. Practitioners may not have been able to adjust to an increasing number of patients with OD due to the time constraints and low profit associated with providing medical care. The mental health of medical professionals themselves may also have been negatively affected by the COVID-19 pandemic.

## Novel Solutions Using Digital Therapeutics

Digital health solutions using web applications are expected to be effective in addressing current challenges and issues surrounding OD ^(5)^. Given that patients with OD may have difficulty attending school or going to the hospital, they may spend a significant amount of time at home on their smartphone screens. Digital therapeutics that support patients with OD and their families have great potential as a novel solution. In particular, established approaches, such as patient education, non-pharmacological interventions, pharmacotherapy, and psychotherapy, can be delivered online. Additionally, patients who regularly visit doctors may be able to utilize mental health care programs provided by clinical psychologists between outpatient visits. In the US, a digital mental health platform has already been launched, offering personalized online mental health care programs for children and their families ^(6)^. These solutions can provide relief for patients and parents, alleviate the stigma surrounding patients, and reduce the burden on health care professionals. It should be noted that digital therapeutics are not meant to replace traditional treatments but rather to supplement them and provide additional support. Patients need to continue receiving care from healthcare professionals, and families also need to discuss the use of digital therapeutics with their doctors. Digital therapeutics can be a valuable resource for managing OD, but it is important to use them as part of a comprehensive treatment plan.

## Future Perspectives

Research on OD has mainly been conducted as a psychosomatic disorder in Japan, while in the US it is classified as a cardiovascular disease characterized by syncope and studied as orthostatic intolerance (OI). The reasons for this difference are that research on OD has advanced in terms of school refusal, which is less common in the US. Moreover, OI is more prevalent among adults; in contrast, OD is a pediatric disorder characterized by difficulty waking up in the morning and associated with sleep disorders. To provide a unique perspective from Japan, it is important to understand the clinical characteristics and epidemiological dynamics of OD due to the COVID-19 pandemic in Japan. For this purpose, further epidemiological studies and random sampling surveys will be needed using large-scale databases in the real world. Additionally, the digital health solutions described in the previous chapter may make it possible to collect more detailed, personalized information. It is also worth considering the potential for international collaboration in researching OD. By bringing together the different perspectives and approaches from Japan and the US, it may be possible to gain a more comprehensive understanding of the disorder and develop more effective treatments.

## Conclusion

In conclusion, the COVID-19 pandemic has exacerbated the situation surrounding OD and limited the effectiveness of existing approaches. Exploring online interventions using digital therapeutics has the potential to be a novel solution for offering necessary support for patients and parents. Additionally, further research utilizing large-scale databases may reveal the clinical epidemiology of OD in the real world, leading to the development of new treatments. Both of these points are urgently needed from a public health perspective, and further advances are expected.

## Article Information

### Conflicts of Interest

None

### Sources of Funding

This work was supported by the Mental Health Okamoto Memorial Foundation.

### Acknowledgement

This work was supported partially by a grant from The Mental Health Okamoto Memorial Foundation. The authors would like to sincerely thank the patients and their parents for sharing their experiences and contributing to the discussion in this work.

### Author Contributions

KS conceptualized the original idea and drafted the initial manuscript. SN critically reviewed and revised the manuscript, and approved the final manuscript as submitted. KS and SN are responsible for all aspects of the work.

### Approval by Institutional Review Board (IRB)

Not applicable
